# The articulatory basis of phonological error patterns in childhood speech sound disorders

**DOI:** 10.3389/fnhum.2025.1635096

**Published:** 2025-10-10

**Authors:** Aravind K. Namasivayam, Leo Yan Li-Han, Jennifer Golabek Moore, Willy Wong, Pascal Van Lieshout

**Affiliations:** ^1^Oral Dynamics Lab, Department of Speech-Language Pathology, University of Toronto, Toronto, ON, Canada; ^2^Department of Surgery, Mayo Clinic, Rochester, MN, United States; ^3^Brave Wings Therapy, Fairfield, NJ, United States; ^4^Department of Informatics, Faculty of Information Science and Electrical Engineering, Kyushu University, Fukuoka, Japan

**Keywords:** speech motor control, phonological error patterns, speech sound disorders, speech development, speech motor delay, articulatory phonology, mutual information analysis, SHAP analysis

## Abstract

Speech acquisition involves complex coordination of articulatory structures, primarily the jaw, lips, and tongue. Typically developing children acquire speech sounds in a hierarchical sequence governed by progressive neuromotor maturation. However, disruptions in speech motor control can lead to systematic phonological error patterns, commonly attributed to cognitive-linguistic deficits. This study explores the articulatory foundations of phonological error patterns in preschool-aged children diagnosed with moderate-to-severe speech sound disorders. Using data from 48 children who participated in a randomized controlled trial, we employed Mutual Information Analysis and Random Forest Models to quantify associations between specific speech motor limitations and phonological error patterns identified via the Diagnostic Evaluation of Articulation and Phonology assessment. Results showed moderate associations between phonological error patterns in particular cluster reduction, final consonant deletion, stopping, gliding, and atypical errors and limitations in jaw, labial-facial, and lingual control. Gliding, unexpectedly, demonstrated a negative relationship with speech motor errors, being more prevalent among older and milder cases, suggesting it may reflect a compensatory developmental stage rather than purely phonological simplification. These findings highlight the significance of speech motor constraints in phonological error patterns, challenging traditional cognitive-linguistic explanations. The results support theoretical frameworks such as Articulatory Phonology, emphasizing that phonological error patterns are influenced substantially by articulatory and neuromotor development. This study underscores the need for integrating motor considerations into clinical assessments and interventions for speech sound disorders in children, fostering a comprehensive approach bridging cognitive-linguistic and motor speech perspectives.

## 1 Introduction

Speech acquisition is a complex process requiring intricate coordination among various articulatory systems, including the jaw, lips, and tongue. Typically developing children master speech sounds through a predictable, hierarchical progression shaped by maturing neuromotor control. Early speech relies heavily on simpler jaw-supported movements, progressing gradually toward refined gestures involving independent movements of the lips and tongue (Davis and MacNeilage, 1995; Locke, 1983; Green et al., 2000, 2002; Kent, 1992). When the development of speech motor control is delayed or disrupted, children may exhibit speech error patterns, such as cluster reductions and other simplifications (Namasivayam et al., 2020). Although psycholinguistic models (e.g., Bradford and Dodd, 1994; Dodd et al., 1989) often attribute these errors solely to cognitive-linguistic (e.g., phonological) deficits, recent evidence suggests a significant contribution from underlying speech motor limitations (Adler-Bock et al., 2007; Gibbon, 1999; Gibbon and Wood, 2002; Gick et al., 2006; Goozée et al., 2007; Namasivayam et al., 2020; Preston et al., 2020; Van Lieshout et al., 2008). Understanding the interplay between phonological errors and speech motor control is essential for accurate diagnosis and targeted intervention in children with speech sound disorders (SSDs). This study explores the direct relationship between specific speech motor limitations and phonological error patterns in preschool-aged children with moderate-to-severe SSDs.

### 1.1 Speech motor development

Speech motor development is sequential; articulators mature at different rates. In early infancy, jaw movements are rudimentary and mostly limited to simple opening and closing movements reflecting an undeveloped ability for precise force control (e.g., Davis and MacNeilage, 1995; Green et al., 2000, 2002; Kent, 1992; Locke, 1983; Namasivayam et al., 2020; Nip et al., 2009; but see Diepstra et al., 2017; Giulivi et al., 2011). During the first year, there is minimal interaction between the lips and the jaw, and the tongue shows little capacity for elevation away from the mandible (Buhr, 1980; Kent, 1992; Otomo and Stoel-Gammon, 1992). By around age two, as coordination between the laryngeal system and oral structures (including the jaw) improves, children begin to exhibit voicing contrasts (Green et al., 2002; Grigos et al., 2005; Yu et al., 2014). At this stage, strong interlip coupling is evident; between ages two and three, this coupling gradually differentiates, allowing the upper and lower lips to move independently, which is critical for the development of labiodental fricatives like /f/ and /v/ (Green et al., 2000, 2002; Green and Nip, 2010; Nip et al., 2009; Stoel-Gammon, 1985).

From ages two to six, lip movements become more finely tuned, and by age three, the tongue starts to operate with increasing independence from the jaw, enabling more accurate anterior–posterior movements (Donegan, 2013; Kent, 1992; Otomo and Stoel-Gammon, 1992; Smit et al., 1990; Wellman et al., 1931). Between three and five years, enhanced coordination between the tongue and jaw emerges, which is essential for articulating more complex sounds (Kent, 1992; McLeod and Crowe, 2018). Because the tongue functions as a hydrostatic organ, achieving fine articulatory coordination depends on both its maturation and exposure to language specific gestural contrasts (Green and Wang, 2003; Kent, 1992; Nittrouer, 1993; Noiray et al., 2013). As the tongue’s various components mature, more intricate sound such as rhotacized vowels and complex fricatives begin to emerge. Ultimately, speech motor variability decreases and stabilizes between the ages of seven and twelve, as coordination among the lips, jaw, and tongue becomes increasingly consistent and efficient (Cheng et al., 2007; Nittrouer, 1993; Nittrouer et al., 1996, 2005; Smith and Zelaznik, 2004; Zharkova et al., 2011; see Figure 1 in Namasivayam et al., 2020).

To summarize, the progression of oral articulatory skills occurs along a variable timeline. The coordination between the lips and the jaw reaches maturity before that of the tongue with the jaw or the independent movement of various tongue segments (Cheng et al., 2007; Terband et al., 2009). Furthermore, speech motor control emerges in a hierarchical, step-by-step, and non-uniform manner, evolving over a prolonged period, which in turn plays a crucial role in acquisition and accurate production of speech sounds (Smith and Zelaznik, 2004; Whiteside et al., 2003). For a comprehensive look at speech motor development see Namasivayam et al. (2020).

### 1.2 Influence of speech motor development on speech sound development

Infants across languages initially produce a limited range of speech sounds and follow a similar order in mastering them, often employing analogous simplification strategies (Green et al., 2002; Locke, 1983; Preisser et al., 1988; Sander, 1972; Stoel-Gammon, 1985). This developmental pattern indicates that emerging speech motor skills most likely influence speech sound acquisition and production (Green et al., 2002). Young children (less than 2 years of age) successfully produce speech sounds that can be effectively formed using the mandible as the primary mover (e.g., /b/), and are less able to produce those that tend to be associated with graded lip control (e.g., /f/).

Further, bilabial stops (/p/, /b/) are highly represented in early phonemic inventories as they can be produced using relatively ballistic jaw movement without active contribution from the lips or tongue (Kent, 1992; MacNeilage and Davis, 1990). On the other hand, sounds like the labiodental fricative /f/, which require fine, independent control of the lower lip and jaw, typically emerge later around 2.5 years of age and are mastered around age 4 (Sander, 1972; Stoel-Gammon, 1985). The preference for jaw-supported sounds in early speech may be attributed to the biomechanical stability of the mandible, a single bone that articulates bilaterally with the temporal bones and is supported by a symmetrical network of muscles (i.e., jaw depressors and elevators), thereby limiting extraneous movements (i.e., limited degrees of freedom in movement directions not related to speech) and providing a stable base for speech (Green et al., 2002; Shiller et al., 2002; Van Lieshout, 2015).

Speech sounds that require even greater finer articulatory control, such as fricatives (/s/), affricates (/t∫/, /dʒ/) laterals and rhotics (/l/, /ɹ/), are mastered later (4+ years) due to the increased demand for independent lip and tongue movements (Kabakoff et al., 2021, 2023; Lin and Demuth, 2015; MacNeilage and Davis, 1990). The control over the interdigitated muscular layers that compose the highly deformable hydrostatic organ, like the tongue, requires relatively greater fine-force regulation and sustained effort over time in comparison with those produced with a more ballistic closure (Abbs et al., 1984; Kabakoff et al., 2021, 2023; Smith and Kier, 1989). These findings indicate that early limitations in speech motor coordination can shape the order in which phonemes are acquired. Green et al. (2000, 2002) highlight that young children’s sound production is restricted by factors such as a reliance on jaw movement, limited coordination between the lips and jaw, limited lip control, and insufficient independent movement of the upper and lower lips, and various functional components of the tongue operating with increasing independence from the jaw (Namasivayam et al., 2020).

Contrary to the above view implicating a stronger role of speech motor development in speech sound acquisition, some researchers have argued that cross-linguistic differences in phoneme error patterns are best explained by factors such as functional load and phonological saliency (Dodd, 2014; Hua and Dodd, 2000). Hua and Dodd (2000) argued that the biological and articulatory constraints observed in young children cannot fully account for these cross-linguistic variations. For instance, they note that the infrequent occurrence of alveolo-palatal affricates in Putonghua (Modern Standard Chinese) and the earlier emergence of affricates in that language compared to English cannot be solely attributed to the frequency of these phonemes in the language or to inherent articulatory constraints (Dodd, 2014). Although the prominence of a phoneme in a child’s native language is important, recent acoustic and transcription findings by Ma et al. (2022) reveal that even within the realm of affricate acquisition in Putonghua language, those that involve the tongue body tend to be mastered earlier than the more complex alveolar and retroflex affricates. This evidence supports the oromotor maturation hypothesis, suggesting that children more readily control the muscles required for elevating the tongue body owing to its earlier development than those needed for raising the tongue tip (Kent, 2021; Li and Munson, 2016; Ma et al., 2022).

Collectively, these findings indicate that early phonological and speech sound development is shaped by several factors, including inherent neuromuscular organization, the spatial and temporal demands of articulating specific phonemes, and influences from the ambient language (Dodd, 2014; Hua and Dodd, 2000; Ma et al., 2022; Namasivayam et al., 2020). Additionally, studies that rely exclusively on auditory-perceptual transcription without instrumental data (e.g., Hua and Dodd, 2000) may have overlooked some of these subtle contrasts due to adults’ categorical perception biases (Kent, 1996; Ma et al., 2022; Meyer and Munson, 2021; Mowrey and MacKay, 1990; Namasivayam et al., 2020). These insights reveal that the process of acquiring speech sounds is more complex than once thought, reflecting a dynamic interaction among cognitive development, ambient language exposure, and oromotor maturation (Green and Nip, 2010; Kent, 2021; Ma et al., 2022; Namasivayam et al., 2020; Nip et al., 2011).

### 1.3 Speech motor development and speech errors: theoretical frameworks

The connection between speech motor development and speech sound errors in children has received limited attention, likely due to the dominance of psycholinguistic models like Dodd’s Model of Differential Diagnosis (MDD; Bradford and Dodd, 1994; Broomfield and Dodd, 2004; Dodd, 2014), which traditionally attribute these errors to phonological factors. Dodd and colleagues integrated the psycholinguistic approach which focuses on input/output processing and internal representations with a clinical descriptive approach based on observed speech errors to develop the foundation for the MDD (Broomfield and Dodd, 2004; Dodd, 2014). Unlike purely developmental models such as Natural Phonology (Stampe, 1969), Dodd’s MDD emphasizes classifying SSDs based on the nature and consistency of error patterns. Within this framework, subgroups are identified according to surface (observed) error patterns, which are believed to reflect distinct underlying deficits in the speech processing chain, including perceptual, cognitive-linguistic, and articulation skills. According to the MDD framework, if a child exhibits phonological error patterns common among typically developing children, albeit with a slight delay, these errors are viewed as part of normal development, and the child is classified as having a “phonological delay.” In contrast, if the phonological error patterns are uncommon, i.e., occur <10% of the time in typically developing children, they are considered atypical, and the child is identified as phonologically disordered (Dodd, 2014).

The MDD model for classifying SSD subtypes is implemented via the Diagnostic Evaluation of Articulation and Phonology test (DEAP; Dodd et al., 2002), which identifies and evaluates ten typical and four atypical phonological error patterns in children. The typical phonological patterns include gliding (e.g., “*rabbit*” → “*wabbit*”), vocalization of liquids (e.g., “*table*” → “*tabo*”), deaffrication (e.g., “*chair*” → “*sair*”), cluster reduction (e.g., “*spoon*” → “*poon*”), fronting (e.g., “*car*” → “*tar*”), weak syllable deletion (e.g., “*banana*” → “*nana*”), stopping (e.g., “*fish*” → “*pish*”), prevocalic voicing (e.g., “*pig*” → “*big*”), postvocalic devoicing (e.g., “*dog*” → “*dok*”), and final consonant deletion (e.g., “*cat*” → “*ca*”). In contrast, atypical phonological patterns include backing (e.g., “*tap*” → “*cap*”), consonant harmony (e.g., “*dog*” → “*gog*”), medial consonant deletion (e.g., “*ladder*” → “*la-er*”), and palatalization (e.g., “*sip*” → “*ship*”). Within the MDD, Dodd (2014) proposes that phonological error patterns typically stem from several sources. These include cognitive-linguistic factors, such as difficulties in learning the phonological rules of a language, or an unstable phonological system. Errors may also arise from anatomical issues (e.g., cleft lip/palate) or muscle function impairments (e.g., Childhood Dysarthria). Additionally, some articulation difficulties, like lisps, occur without an identifiable cause and are believed to result from mislearning the mapping between perceptual and articulatory systems.

In contrast to Dodd’s MDD, linguistic theories such as Natural Phonology (Stampe, 1969), Grounded Phonology (Archangeli and Pulleyblank, 1994), and Articulatory Phonology (Browman and Goldstein, 1992; Namasivayam et al., 2020; Van Lieshout and Goldstein, 2008) all explicitly acknowledge the influence of phonetic and articulatory factors on phonological processes, a perspective not emphasized in Dodd’s MDD. These theories recognize that ease of articulation and perceptual clarity play significant roles in shaping sound patterns across languages. While Natural Phonology focuses on developmental processes and innate simplification strategies in speech, Grounded Phonology adopts a more formalist, system-wide approach, integrating phonetics and phonology within constraint-based frameworks, often aligned with Optimality Theory. It explains phonological markedness through universal phonetic motivations, suggesting that certain sounds are avoided across languages due to articulatory complexity or perceptual ambiguity.

Articulatory Phonology (AP), as outlined by Namasivayam et al. (2020), provides a dynamic approach to understanding speech sound disorders in children, focusing on the role of articulatory gestures which control coordinated movements of the speech organs in both typical and disordered speech production. This framework suggests that SSDs often stem from disruptions in the planning and execution of these gestures, leading to observable speech errors. Importantly, Namasivayam et al. (2020) posits that many of these errors may not solely represent deficits but also serve as compensatory strategies aimed at enhancing speech motor stability. Such adaptations include increased movement amplitude, slower speech rates, tongue bracing, intrusion gestures, cluster reduction, and deletions at the segmental, gestural, or syllabic levels. Additionally, increased phase lag between articulatory movements has been observed as a means of improving motor stability and intelligibility, particularly in speakers with less developed speech motor skills (Fletcher, 1992; Namasivayam et al., 2020; Van Lieshout et al., 2004). This integrative framework combines elements of speech perception, motor execution, and neural control, offering a comprehensive perspective for understanding and addressing SSDs. While Grounded Phonology focuses on explaining phonological patterns through universal phonetic constraints and articulatory ease, Articulatory Phonology emphasizes the real-time coordination and motor processes involved in speech production. This allows for a more detailed exploration of how breakdowns in gestural coordination contribute to speech errors, highlighting the complex interplay between motor control and phonological output in both typical and disordered speech (Namasivayam et al., 2020; Van Lieshout and Goldstein, 2008).

Notably, within the AP model articulatory gestures are defined at an abstract cognitive level by parameters related to constriction location and degree and by their temporal organization. They function as the speech system’s basic phonological primitives. Gestures combine, overlap, or are withheld to create contrast at the level of segments, words, and larger utterances. Linguistic phonological contrast can arise from the presence/absence of a gesture or from parametric differences within the same gesture. For example, “*ban*” differs from “*bad*” by the addition of a velic-lowering (nasal) gesture; “*bad*” versus “*pad*” is distinguished by a laryngeal gesture supporting voicing in the former; and “*bus*” versus “*but*” reflects a difference in tongue-tip constriction degree (narrow, frication-supporting aperture for /s/ vs. complete closure for /t/). Each gesture has an internal temporal profile with landmarks (onset, target, release), and the alignment of these landmarks across gestures yields the observable segmental structure and higher-level units. These patterns of gestures and their timing relations are typically schematized as a gestural score (Browman and Goldstein, 1990, 1992; Van Lieshout et al., 2008). Thus, within the AP framework, gestures are not defined at the articulatory level (level of execution) but rather at the cognitive stages of speech production (e.g., Goldstein et al., 2006, 2007). In this account, those gestures and subsequent stages in the model substitute for theoretical concepts of phonemes and features as used in more traditional psycholinguistic theories.

Let’s briefly look at potential mechanisms for a speech motor basis of so-called “phonological” process errors.

### 1.4 Potential speech motor basis for “phonological” process errors

Given prior evidence on speech motor development, young children’s motor limitations are likely to contribute to systematic speech errors. In early speech development the reliance on ballistic jaw movements may mean that certain ‘phonological’ error patterns, such as substitutions and omissions, are more likely to occur in sounds requiring graded control (Kent, 1992; Tobin, 1997). In two-year-olds, the upper and lower lips typically move in unison with the jaw rather than independently. At this stage, a child might correctly produce /f/ only in jaw-supported contexts such as in transitions from an open-to-closed position (e.g., “*off*”) or closed-to-open (e.g., “*fan*”) but struggle with words like “*fit*” or “*fish*”, where the jaw remains in a stable, high-vowel position. This limitation can result in substitution errors, such as producing “*pish*” for “*fish*”. Difficulties with labiodental fricatives in early speech may also arise from the child’s inability to sustain airflow and control lip movements with the precision required for accurate production (Namasivayam et al., 2020; Tobin, 1997).

Similarly, children under the age of three, especially those with SSDs, often exhibit undifferentiated tongue gestures, i.e., limited independent tongue-tip elevation (Gibbon, 1999). As a result, alveolar consonants are frequently produced using jaw-supported tongue elevation (often observed as the jaw and tongue moving in the same direction, i.e., in-phase movements), creating what is known as jaw-compensated speech. In this synergy between the jaw and tongue tip, the jaw aids in achieving the necessary tongue-tip elevation for alveolar sounds. Thus, it is not surprising that the SSD population also has a high prevalence of (anterior or lateral) jaw sliding, likely representing an adaptive jaw response to reduced tongue control (Mogren et al., 2020, 2022; Namasivayam et al., 2013, 2020; Terband et al., 2013).

Such an adaptive jaw response to tongue movement limitations has also been reported in the adult neurodegenerative literature (Rong and Green, 2019). In the early stages of bulbar Amyotrophic Lateral Sclerosis (ALS), as tongue control deteriorates and speech intelligibility decreases, jaw-supported tongue movements have been observed as a compensatory strategy to counteract this decline in intelligibility. However, as bulbar ALS progresses, this adaptive jaw strategy diminishes, leading to a further reduction in speech intelligibility (Mefferd and Dietrich, 2020; Rong and Green, 2019). Furthermore, while the jaw-compensated speech strategy may help maintain intelligibility at the word level, it can reduce clarity in connected speech due to slower movements of the jaw (Green et al., 2000, 2002).

Thus, common phonological error patterns, such as cluster reduction, fronting, and stopping, can be attributed to issues with the ongoing refinement of speech motor control and the development of articulatory coordination. Additionally, errors that affect syllable and word shapes factors known to significantly impact speech intelligibility (Hodge and Gotzke, 2011; Osberger and McGarr, 1982) are likely rooted in the constraints of an immature oromotor system, as well. Next, we show how motor constraints may underlie DEAP “phonological” processes (Dodd et al., 2002), elaborated in Namasivayam et al. (2020).

#### 1.4.1 Stopping

Stop substitution of fricatives may arise from an inappropriate specification of constriction degree (Constriction Degree: /d/ closed vs. /z/ critical; Goldstein et al., 2006), likely a simplification process arising from limited precision of tongue tip control (Tobin, 1997).

#### 1.4.2 Gliding and vocalization of liquids

Gliding involves substituting a liquid sound with a glide, such as “rabbit” /ræbɪt/ becoming [wæbɪt] while vocalization of liquids occurs when a liquid is replaced with a vowel, like “apple” /æpl/ → [æp℧] (McLeod and Baker, 2017). The /r/ sound is acoustically marked by a drop in the third formant (Alwan et al., 1997) and is kinematically complex, involving coordination of at least three gestures viz., the lips, tongue tip/body, and tongue root (Adler-Bock et al., 2007; Gick and Campbell, 2003; Preston et al., 2020). Due to its complexity, /r/ is typically mastered between ages 4 and 7 (McLeod and Crowe, 2018). Ultrasound studies suggest that children struggle with coordinating these gestures, often simplifying /r/ by omitting one gesture, leading to errors (Adler-Bock et al., 2007; Preston et al., 2020). Syllable-final /r/ sounds are frequently vocalized because the resulting articulation retains only some of the original constrictions, leading to a more vowel-like quality (Adler-Bock et al., 2007). For example, a child may omit the tongue-tip gesture but retain lip rounding, which then dominates the acoustic output (Adler-Bock et al., 2007; Van Lieshout et al., 2008). Electromagnetic articulography data supports this, revealing limited differentiation between tongue parts and timing issues in /r/ errors (Van Lieshout et al., 2008).

#### 1.4.3 Velar fronting and coronal backing

Velar fronting is characterized by substituting sounds produced at the back of the vocal tract with those articulated further forward, such as replacing /k/ with /t/ (e.g., “key” → [ti]; McLeod and Baker, 2017). Coronal backing, conversely, involves replacing front sounds with back ones (e.g., “two” → [ku]; McLeod and Baker, 2017). These errors are often associated with undifferentiated lingual gestures, where tongue movements lack clear coordination between the tip, body, dorsum and lateral margins (Gibbon, 1999). Electropalatography (EPG) and electromagnetic articulography studies show that, instead of focused anterior contact for alveolar sounds, tongue-palate contact extends into palatal or velar areas (Gibbon and Wood, 2002). Approximately 71% of children aged 4–12 with articulation and phonological disorders display these undifferentiated gestures (Gibbon, 1999). Such patterns may result from decreased oromotor control, immature speech motor systems, or compensatory strategies to stabilize tongue movements (Goozée et al., 2007). Standard acoustic-perceptual transcription often fails to detect these gestures, leading to inconsistent classifications as distortions, substitutions, or correct productions (Gibbon, 1999; Gibbon and Wood, 2002). The perceived articulation site is influenced by articulatory drift during tongue-palate contact (Gibbon and Wood, 2002).

#### 1.4.4 Prevocalic voicing and postvocalic devoicing

Prevocalic voicing and postvocalic devoicing are common childhood speech errors in which syllable position conditions consonant voicing. Prevocalic voicing occurs when voiceless consonants in syllable-initial positions are replaced by voiced ones (e.g., “pea” /pi/ → [bi]), while postvocalic devoicing substitutes voiced consonants in syllable-final positions with voiceless ones (e.g., “bag” /bæg/ → [bæk]; McLeod and Baker, 2017). These patterns are tied to the complexity of coordinating articulatory gestures (see Namasivayam et al., 2020 for detailed overview). In syllable onsets, gestures like bilabial closure and glottal gestures for voicing occur in-phase, forming a stable configuration that facilitates voicing (Goldstein et al., 2006; Krakow, 1993). In contrast, coda positions require asynchronous gestures, increasing motor demands and often leading to devoicing (Goldstein et al., 2006; Haken et al., 1985). Jaw control also influences voicing accuracy, as precise timing between glottal and oral gestures is key to voice-voiceless contrasts. Increased jaw movement and stability enhance voicing control in young children (Grigos et al., 2005). Children with SSDs often exhibit jaw instability, disrupting voicing contrasts (Namasivayam et al., 2013; Terband et al., 2013). Jaw stabilization interventions have shown to improve voice onset times, especially for sounds like /p/ (Yu et al., 2014). Since the perioral area lacks certain sensory receptors, reliable feedback for coordinating laryngeal and oral gestures may come from jaw masseter muscle spindles (Namasivayam et al., 2009; Van Lieshout, 2015). Thus, enhanced jaw stability provides consistent feedback, supporting better integration of glottal and oral movements for accurate voicing (Namasivayam et al., 2009; Van Lieshout, 2017; Yu et al., 2014).

#### 1.4.5 Final consonant deletion

Final consonant deletion in children, where the final consonant of a word is omitted (e.g., “cat” /kæt/ → [kæ]), can be explained by challenges in speech motor coordination and stability (Namasivayam et al., 2020). According to the articulatory phonology (AP) framework, consonant-vowel (CV) sequences are produced in a stable, in-phase manner, making them easier to coordinate than vowel-consonant (VC) or consonant-consonant (CC) sequences, which require less stable, anti-phase coordination (Giulivi et al., 2011; Goldstein et al., 2006; Nam et al., 2009). As children’s cognitive-linguistic and motor demands increase, maintaining the less stable CVC structure becomes difficult, leading to the deletion of final consonant to preserve the more stable CV structure (Goldstein et al., 2006). Ultrasound studies have also shown that shared coda consonants can destabilize speech motor patterns, increasing the likelihood of errors like final consonant deletion (Mooshammer et al., 2019; Pouplier, 2008). Additionally, limited jaw control can cause final consonant deletion, especially when close–open–close sequences tax precise jaw elevation (Namasivayam et al., 2020). Rather than indicating a phonological disorder, these deletions often reflect compensatory strategies to manage an unstable speech motor system.

#### 1.4.6 Weak syllable deletion

Weak syllable deletion involves omitting an unstressed syllable in a word, such as “banana” /bənænə/ → [nænə] (McLeod and Baker, 2017). Tilsen (2009) explains the deleting an unstressed syllable in a multisyllabic word as a strategy to reduce complexity of coordination between syllable-level and stress-level neuronal populations (within the AP model these are referred to as neuronal oscillators; Goldstein et al., 2007). Deleting syllables allows the speech motor system to operate in a more stable state (See Namasivayam et al., 2020 for more information).

#### 1.4.7 Cluster reduction

Cluster reduction involves the omission of a consonant from a cluster, often the more marked one, simplifying words like “please” /pliz/ to [piz], “blue” /blu/ to [bu], and “spot” /spɒt/ to [pɒt] (McLeod and Baker, 2017). From a motor stability standpoint, CC onset clusters are less stable (anti-phasic) and, under increased speech motor demands or immaturity (Fletcher, 1992), are often reduced to a simpler CV structure by omitting an extra consonant gesture (Goldstein et al., 2006; Nam et al., 2009; Van Lieshout and Goldstein, 2008). Another explanation is gestural hiding, where overlapping gestures in heterorganic clusters can obscure one consonant acoustically and perceptually (Browman and Goldstein, 1990; Gibbon et al., 1995; Hardcastle et al., 1991).

These findings indicate that early limitations in speech motor coordination can shape the order in which phonemes are acquired and executed. Green et al. (2000, 2002) highlight that young children’s sound production is restricted by factors such as a reliance on jaw movement, limited coordination between the lips and jaw, limited lip control, and insufficient independent movement of the upper and lower lips, and various functional components of the tongue operating with increasing independence from the jaw (Namasivayam et al., 2020).

## 2 The current study

Evidence supports a strong relationship between speech motor control and speech intelligibility (Namasivayam et al., 2013), as well as links between segmental errors and intelligibility in groups such as hearing-impaired children (Hodge and Gotzke, 2011; Osberger and McGarr, 1982). However, the specific role of motor limitations in shaping speech-sound errors in children with SSDs remains underexplored. Preliminary instrumental findings across two SSD subtypes—persistent speech sound disorder (Preston et al., 2020) and articulation/phonological disorder (Gibbon, 1999; Gibbon and Wood, 2002) associate “phonological” error patterns with articulatory constraints. Although theories such as Natural Phonology, Grounded Phonology, and Articulatory Phonology implicitly posit motor influences on phonological development, empirical data directly linking motor control to “phonological” patterns (e.g., those indexed by the DEAP assessment; Dodd et al., 2002) in children with SSDs remain limited. This gap motivates further study of how motor control, articulation, and phonological development interact. In our manuscript, we focus on children classified with the Speech Motor Delay (SMD) subtype to reduce phenotypic heterogeneity and test whether specific phonological error patterns covary with measurable speech motor limitations. We ask whether, within an a priori group with known motor vulnerabilities, the distribution of error patterns typically associated with phonological processes systematically aligns with motor constraints. By examining these relationships, we aim to clarify mechanisms underlying speech-sound acquisition and error formation in SSDs, thereby informing more effective assessment and intervention strategies (McLeod and Baker, 2017).

This study presents data from 48 preschool-age children who participated in a recently completed randomized controlled trial (RCT; Namasivayam et al., 2021a; Namasivayam et al., 2021b). The comprehensive pre-treatment assessments conducted as part of the RCT provide a unique opportunity to investigate the relationship between “phonological” error patterns, as identified in the DEAP assessment (Dodd et al., 2002), and speech motor characteristics observed in children with SSDs. Specifically, we aim to assess the association between speech motor control characteristics, such as lateral jaw sliding, inadequate integration of jaw and lips, and limited tongue tip elevation from the jaw as proposed by Green et al. (2000, 2002) (see Methods for further details; Namasivayam et al., 2013; Namasivayam et al., 2021a; Namasivayam et al., 2021b) and the types of errors observed in the DEAP phonological assessment (Dodd et al., 2002). We hypothesize a non-zero (i.e., non-independent) relationship between these variables, supporting theoretical frameworks such as natural phonology, grounded phonology, and articulatory phonology. In other words, we hypothesize that specific speech motor control limitations are significantly associated with, and can predict, the occurrence and type of phonological error patterns in preschool-aged children with moderate-to-severe SSDs. To quantify the association between phonological error patterns and speech motor characteristics, we employed statistical approaches, including Mutual Information Analysis and Random Forest Models, commonly used in machine learning research (Breiman, 2001; Scott and Su-In, 2017; Strehl and Ghosh, 2002).

### 2.1 Research question

To what extent are specific speech motor control limitations (e.g., lateral jaw sliding, limited tongue tip elevation from the jaw, inadequate jaw–lip integration) associated with, and predictive of, the occurrence and type of phonological error patterns (as identified by the DEAP) in preschool-aged children with moderate-to-severe SSDs?

## 3 Methods

### 3.1 Participants and setting

This study analyzed pre-treatment data from 48 children (mean age: 48 months, SD = 11) drawn from a larger randomized controlled trial on speech motor intervention (Namasivayam et al., 2021a; Namasivayam et al., 2021b). Participants were recruited from community-based healthcare centers in Mississauga, Toronto, and Windsor, Ontario, Canada. Demographic details are presented in Table 1. Children were eligible if they met the following criteria: (1) age between 3 and 10 years, (2) English as the primary language spoken at home *(language background data collected did not extend beyond the English-speaking requirement; we did not gather detailed information on additional languages the child may have been exposed to or using)*, (3) diagnosis of moderate-to-severe SSD, specifically an SMD subtype, based on features reported in the precision stability index (Shriberg et al., 2010; Shriberg and Wren, 2019), (4) normal hearing and vision *(as confirmed by parent reports and school records)*, (5) Primary Test of Nonverbal Intelligence score at or above the 25th percentile with a standard score of ≥90 (Ehrler and McGhee, 2008), (6) receptive language standard score of ≥78 on the Clinical Evaluation of Language Fundamentals (Semel et al., 2003, 2004), (7) no restrictions on expressive language scores, (8) age-appropriate social skills, (9) age-appropriate play skills, (10) readiness for therapy: presence of intentional communication, (11) readiness for therapy: ability to imitate, (12) behaviourally ready for therapy and (13) presence of at least four motor speech limitation indicators as identified in the motor speech checklist (item 13–21; See Table 2 below; Namasivayam et al., 2013; Namasivayam et al., 2021a; Namasivayam et al., 2021b).

**TABLE 1:** Participant demographics.

Variable	Mean (*SD*) or count (%)
Participants	*N* = 30
Age in months	51 (13)
Gender	Female = 10, Male = 20
Primary language (English) spoken at home	30 (100%)
Hearing and vision (within normal limits)	30 (100%)
History of speech and language intervention	20 (66%)
Primary test of nonverbal intelligence^a^ nonverbal index standard score	102 (21)
VMPAC focal oromotor and sequencing subsections^b^ (%)	67 (13)
DEAP phonological assessment – standard score^c^	61 (6)
DEAP inconsistency assessment (%)^c^	33 (19)
Percent consonants correct (%)^c^	41 (19)
Percent vowels correct (%)^c^	84 (10)
Clinical Evaluation of Language Fundamentals^d^	
• Receptive language index standard score	96 (14)
• Expressive language index standard score	77 (15)
Children’s speech intelligibility measure (word-level %)^e^	41 (17)
Beginners intelligibility test (sentence-level %)^f^	20 (16)
Focus on the outcomes of communication under six^g^	221 (45)
Motor speech checklist (max score = 18)^h^	16.5 (1.6)
Childhood apraxia of speech checklist (max score = 12)^i^	5 (1)

*^a^*Primary test of nonverbal intelligence (Ehrler and McGhee, 2008).

*^b^*Verbal Motor Production Assessment for Children (VMPAC; Hayden and Square, 1999).

*^c^*Phonological assessment, Inconsistency assessment, Percentage of Consonants Correct (PCC) and Percentage of Vowels Correct (PVC) (Shriberg et al., 1997) extracted from the Diagnostic Evaluation of Articulation and Phonology Test (DEAP; Dodd et al., 2002).

*^d^*Standard scores of clinical evaluation of language fundamentals (CELF-4, Semel et al., 2003; CELF Preschool-2, Semel et al., 2004).

*^e^*Children’s speech intelligibility measure (CSIM; Wilcox and Morris, 1999).

*^f^*Beginners intelligibility test (Osberger et al., 1994).

*^g^*Focus on the outcomes of communication under six (FOCUS-50; Thomas-Stonell et al., 2010).

*^h^*Motor speech checklist (Namasivayam et al., 2013).

*^i^*Childhood apraxia of speech checklist (Namasivayam et al., 2015).

**TABLE 2:** Indicators of motor speech limitations or red flags for motor speech issues in children used as study inclusion criteria.

Speech motor control characteristics	
**13. Jaw control**	
Jaw 13.1	Inadequate jaw opening (over extension / too restricted)
Jaw 13.2	Inability to grade jaw for mid-height vowels. /I, e, є, Υ, o, λ/
Jaw 13.3	Decreased jaw stability/ decreased mid-line jaw control (lateral/anterior jaw sliding)
**14. Labial-facial control**	
Lip face 14.1	Inadequate bilabial contact for /p, b, m/ with any vowel.
Lip face 14.2	Non-independent bilabial movement from jaw for /p, b, m/ to/from high vowels /i, I, e, u/ and contribution of upper/lower lips unequal e.g. “beep”
Lip face 14.3	Lower lip movement for /f/, is not independent from jaw e.g. “feet”
Lip face 14.4	Inadequate lip rounding for /o/ and /u/ (no jaw help) e.g. “no”, “boot”
Lip face 14.5	Inadequate lip retraction for /i/, /e/ (symmetrical with no “fixing” at lip corners)
**15. Integration of Jaw and Lips (2-plane movements)**	
Integrate 15.1	Jaw and Lips - Inadequate jaw range with lip rounding for /au/ e.g. “down”
Integrate 15.2	Jaw and Lips - Inadequate jaw range with lip retraction for /ai/ or across 2 syllables e.g. “bite”, “mommy”
**16. Lingual control**	
Lingual 16.1	Non-independent tongue tip elevation from jaw for /t, d, n/ e.g. “two”, “no”
Lingual 16.2	Inaccurate posterior movement (velar /k/ and /g/ e.g. “cookie”, “go”)
**17. Multi-plane movements**	
Multiplane 17.1	Inability to alternate lip retraction with lip rounding e.g. “yoyo”
Multiplane 17.2	Inability to produce multi-syllabic words with change of place and plane of movement e.g. “ladybug”, “doubleyou”
**General speech production characteristics**	
18 Limited variety	Limited variety of speech motor movements (e.g. uses jaw as primary articulator, ie. jaw-supported speech).
19 Limited vowel	Child presents with limited vowel repertoire and/or vowel distortions or a limited consonant repertoire and/or consonant distortions.
20 Limited shapes	Child presents with limited syllable and word shapes
21 Length complexity	Child presents with difficulty maintaining sound and syllable integrity with increased length and complexity of utterance.

Items 1–12 relate to general inclusion criteria and items 13–21 identify motor speech limitations. Namasivayam et al. (2013), Namasivayam et al. (2021a), Namasivayam et al. (2021b).

Children were excluded if they exhibited any of the following: (1) signs of global motor involvement (e.g., cerebral palsy), (2) more than seven out of 12 indicators on a CAS checklist (American Speech-Language-Hearing Association, 2007; cutoff from Namasivayam et al., 2015), (3) feeding difficulties, drooling, or oral structural/resonance issues, and (4) a diagnosis of autism spectrum disorder. A licensed SLP conducted all assessments for inclusion and exclusion criteria. The study received approval from the University of Toronto’s research ethics board (Protocol 29142), with additional approvals from participating clinical sites.

### 3.2 Data collection, recording and reliability

All assessment and intervention sessions were recorded in both video (JVC Everio GZ-E220 HD, 1,920 × 1,080 resolution) and audio (Zoom H1 Ver 2.0, 16-bit at 44.1 kHz) formats for inter-rater reliability analysis. κ-Statistics was used to measure agreement, with values categorized as poor (<0), slight (0.2), fair (0.4), moderate (0.6), substantial (0.8), and almost perfect (1) (Viera and Garrett, 2005). The κ coefficient, calculated from 20% of the data by blinded SLPs, averaged 0.73 (substantial) for auditory-perceptual (International Phonetic Alphabet) transcriptions for the DEAP assessments (Dodd et al., 2002). The reliability of observing speech motor control deficits across different articulators using specialized probe words (Namasivayam et al., 2021a) ranged from fair to moderate: mandibular (0.52), labial–facial (0.57), lingual (0.63), and sequenced items (0.48). κ-statistics for intra- and inter-rater reliability (calculated on 20% of randomly sampled data) for the motor speech checklist was 0.65 and 0.54, respectively. Note: The SLP who completed the motor speech checklist at study inclusion used different assessment methods (spontaneous speech sample, articulation testing, and a live sample) than the SLP who later scored the checklist using recorded probe words. Consequently, inter-rater reliability was somewhat lower than intra-rater reliability, as expected.

All assessments were carried out by licensed speech-language pathologists in a quiet room, with age-appropriate decor and stimuli using standardized testing procedures reported in the literature (Namasivayam et al., 2013; Namasivayam et al., 2021a; Namasivayam et al., 2021b). Furthermore, samples were also excluded from the analysis if there were missing data in either phonological or speech motor control records. In total, data from 30 children were included in the analysis after exclusion.

### 3.3 Data analysis

We conducted Mutual Information Analysis, a Random Forest model, and additional correlational analyzes (Pearson’s *r*) to interpret the findings, along with one-way ANOVA and Kruskal-Wallis tests to assess statistical significance. Given the high linguistic diversity of the recruitment regions, it is likely that some children were bilingual or multilingual; however, subgroup analyzes examining the effects of linguistic background on motor control were not feasible due to the small sample size and the wide variety of potential language pairings.

#### 3.4 Mutual information analysis

Mutual Information (MI) analysis was used to quantify the association between phonological error types from the DEAP assessment (Dodd et al., 2002) and limitations in speech motor control (checklist, see Table 2). MI is a non-parametric measure that captures the dependency between two variables by quantifying the reduction in uncertainty of one variable given knowledge of the other (Cover and Thomas, 1999). Unlike linear correlation measures such as Pearson or Point-Biserial correlation coefficients, MI does not assume a specific functional form of the relationship. This is important in that phonological and speech motor mechanisms have complex and potentially non-linear relationships. Mutual information can handle both normal and non-normal distributions and is a robust measure of association between different non-linear variables (Breiman, 2001; Scott and Su-In, 2017; Strehl and Ghosh, 2002).

The mutual information between each phonological error *X* and each speech motor error *Y* is denoted as *MI* (*X*, *Y*) which we define as follows:


(1) M⁢I(X,Y)=H(X)-H(Y)=H(X)+H(Y)-H(X,Y)

$$=\underset{x\in X}{\sum }\underset{y\in Y}{\sum }p\left(x,y\right)log\frac{p\left(x,y\right)}{p\left(x\right)p\left(y\right)}$$

where *H* (*X*) and *H* (*Y*) represent the marginal entropy of random variables *X* and *Y*, respectively, *H* (*Y*) is the conditional entropy of *X* given *Y*, *H* (*X*, *Y*) represents the joint entropy of *X* and *Y*, *p* (*x*, *y*) is the joint probability distribution of *X* and *Y*, and *p* (*x*) and *p* (*y*) are marginal probability distributions of *X* and *Y*, respectively.

The quantity expressed in Equation 1 is a non-negative and it gives a symmetrical measure of the information shared between *X* and *Y*, with *MI* (*X*, *Y*) = 0 when the two variables are statistically independent. Also, higher MI values indicate stronger associations between phonological and speech motor limitations, suggesting that specific phonological errors may be correlated with certain speech motor impairments.

Furthermore, considering the differences in scale and measurement between phonological and speech motor limitations, we normalized MI scores to facilitate interpretation and comparison across different feature associations. Specifically, we used the geometric mean normalization method introduced by Strehl and Ghosh (2002), which accounts for entropy in both variables and is defined as follows:


(2) $$N\,M\,I\left(X,Y\right)=\frac{M\,I\,\left(X,Y\right)}{\sqrt{H\,\left(X\right)\,H\,\left(Y\right)}}$$

where *H* (*X*) and *H* (*Y*) represent the marginal entropy of the phonological error *X* and speech motor limitation *Y*, respectively. This formulation ensures that the normalized MI (NMI) value remains within the range [0,1], making it more interpretable and similar to the Pearson Correlation Coefficient (Lange and Grubmüller, 2006).

Since there is no universal guideline for interpreting NMI, we employed interpretation conventions from previous literature on correlation coefficients to analyze the NMI values (Overholser and Sowinski, 2008; Schober et al., 2018). Specifically, in this study, the association between phonological and speech motor limitations is considered weak (*NMI* < 0.2), fair (0.2 ≤ *NMI* < 0.4), moderate (0.4 ≤ *NMI* < 0.7), and strong (*NMI* ≥ 0.7), respectively.

We have excluded total speech motor checklist scores, which represents the arithmetic sum of all speech motor limitations (items 13 – 21 – Table 2) from the MI analysis, as it is different from the other motor features which are binary in their outcome space. Additionally, one sample with a significantly lower motor checklist score (lower than the 3x standard deviation range) was considered an outlier and was also excluded from the analysis.

#### 3.4 1 Random forest model for predicting total motor errors

As the total speech motor checklist scores were not used in the MI analysis above, we further trained a Random Forest (RF) model to predict the motor checklist score (i.e., the overall speech motor control performance) from phonological error features. This aimed to investigate the importance (or predictivity) of phonological error patterns in estimating speech motor characteristics, thereby revealing the association between these two feature types. Random Forest is an ensemble machine learning method that constructs multiple decision trees and aggregates their predictions, enhancing model robustness and reducing variance (Breiman, 2001). It is well-suited for our study due to its ability to handle both linear and non-linear relationships and its robustness to overfitting, particularly in scenarios with small sample sizes.

During training, the RF model was optimized using a randomized search approach, where hyperparameters such as the number of trees, maximum depth of trees, and the minimum number of samples per split were tuned by randomly sampling from a specified range, instead of performing an exhaustive grid search, which would be computationally prohibitive even for small datasets.

To ensure generalizability of our trained RF model, we employed Leave-One-Out Cross-Validation (LOOCV), which is a validation strategy particularly suitable for small datasets. In LOOCV, the model was trained on *N*−1 samples and tested on the held-out sample, repeating this process *N* times (where *N* is the total number of samples). The final performance of the RF model was assessed by averaging the evaluation metrics (i.e., the Mean Absolute Error) across all cross-validation folds. LOOCV provides an unbiased estimate of model performance and maximizes the use of available data for both training and testing, making it ideal given our sample size.

With the trained RF model, we investigated the importance of phonological features in predicting the total motor error score using the SHapley Additive exPlanations (SHAP), a method derived from cooperative game theory (Scott and Su-In, 2017) and has widely used for machine learning model interpretation. SHAP values provide a directional measure of feature importance by quantifying the marginal contribution of each predictor to the model’s output, which offers a more interpretable decomposition of the prediction and the relative contribution between different predictor variables. As such, we utilized SHAP analysis to compare the relative, directional influence of those phonological error patterns, while the NMI values from mutual information analysis were used to interpret the strength of association.

Specifically, we visualized the feature importance using a SHAP bar plot and swarm plot. The bar plots displayed the mean absolute SHAP values across all samples, ranking phonological features in order of their influence on motor error predictions. The swarm plots further illustrated the distribution of SHAP values for each feature, providing insights into how specific feature values contributed to predictions across different samples.

## 4 Results

The results show that, in our dataset, CC REDUCTION (cluster reduction), FCD (final consonant deletion), STOPPING, Atypical Patterns, and GLIDING were the top 5 common phonological error patterns, with the mean (standard deviation, STD) frequency/count of 16.6 (5.5), 12.8 (11.6), 12.7 (8.1), 8.8 (8.3), and 8.5 (6.1), respectively. Meanwhile, different types of speech motor control limitations distributed relatively uniformly across the patients, with a mean (STD) total checklist score of 16.5 (1.6). The detailed data characteristics of phonological error and total motor checklist score data are summarized in Table 3.

**TABLE 3:** Data statistics of phonological error patterns and total speech motor checklist scores.

Phonological error pattern	Mean (standard deviation)	Median (interquartile range)
Gliding	8.5 (6.1)	7.5 (3.2–13.0)
Vocalization of liquids	4.8 (2.5)	5.0 (3.2–6.8)
Deaff	0.4 (0.8)	0 (0–0.8)
CC reduction	16.6 (5.5)	17.0 (12.2–20.8)
Fronting	7.3 (5.4)	6.0 (3.2–10.8)
WSD	2.7 (2.2)	2.0 (1.0–4.0)
Stopping	12.7 (8.1)	11.0 (6.0–19.2)
Pre-vocalic voicing	6.2 (7.4)	2.5 (0.2–10.0)
Post-vocalic devoicing	1.1 (1.5)	0.5 (0.0–1.8)
FCD	12.8 (11.6)	8.5 (2.5–23.8)
Atypical patterns total	8.8 (8.3)	5.5 (3.2–11.0)
**Speech motor control errors**		
Jaw control	2.9 (0.4)	3.0 (3.0–3.0)
Labial-facial control	4.2 (1.1)	5.0 (3.2–5.0)
Integration of jaw and lips	2.0 (0.2)	2.0 (2.0–2.0)
Lingual control	1.5 (0.6)	2.0 (1.0–2.0)
Multi-plane movements	2.0 (0.2)	2.0 (2.0–2.0)
General speech production characteristics	4.0 (0.2)	4.0 (4.0–4.0)
Total motor checklist score	16.5 (1.6)	17.0 (15.0–18.0)

### 4.1 Mutual information (MI) analysis

**FIGURE 1:**
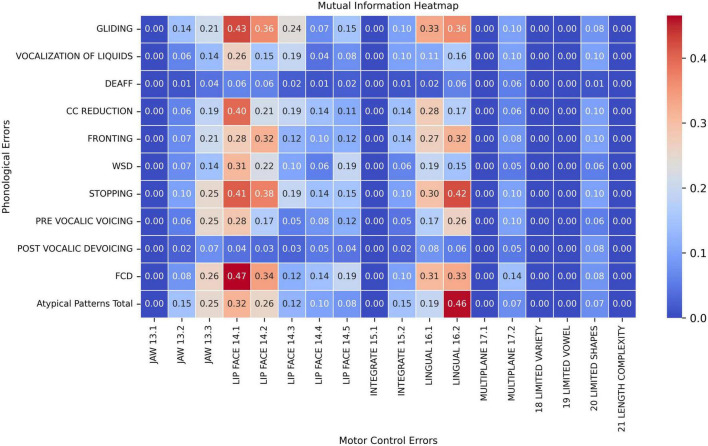
Pair-wise mutual information heatmap between speech motor control limitations (x-axis) and phonological error patterns (y-axis).

Figure 1 illustrates the pair-wise mutual information heatmap graph between speech motor control limitation (x-axis) and phonological error pattern (y-axis), with warmer colors (red) indicating stronger associations and the number within each cell the mutual information (NMI) of the feature pair. Specifically, phonological errors were primarily associated with three types of speech motor control limitations: labial-facial control (items 14.1 and 14.2 in Table 2), lingual control (items 16.1 and 16.2), and, to a lesser extent, jaw control (item 13.3). These findings are generally consistent with those proposed by Green et al. (2000, 2002) who identified jaw control, integration of the jaw and lips, upper–lower lip movement independence, and tongue–jaw dissociation as critical for accurate speech sound production in young children. In contrast, other speech motor limitations, including Multi-plane Movements (items 17), General Speech Production Characteristics (items 18– 21), and Integration of Jaw and Lips (items 13), exhibited only limited association with phonological errors (NMI < 0.20).

When examining phonological error patterns, FCD (final consonant deletion), GLIDING, and STOPPING demonstrated fair to moderate associations with both Labial-Facial and Lingual control limitations (items 14.1, 14.2, 16.1, and 16.2; NMI > 0.30). Meanwhile, the CC REDUCTION (cluster reduction) error only showed stronger association with speech motor control item LIP FACE 14.1 (NMI = 0.40). Among all feature pairs, phonological error FCD and motor control limitation LIP FACE 14.1 exhibited the highest connection with an NMI value of 0.47 (moderate association). On the other hand, phonological errors such as DEAFF (deaffrication), PRE-VOCALIC VOICING, POST-VOCALIC DEVOICING, and VOCALIZATION OF LIQUIDS demonstrated weaker associations with motor control errors, with the NMI below 0.20 across most motor control categories. Additionally, atypical phonological errors also presented stronger associations with posterior movement-related lingual control errors (LINGUAL 16.2, NMI = 0.46). Lastly, Atypical Patterns, FCD, PRE-VOCALIC VOICING and STOPPING had fair associations with Jaw sliding (anterior/lateral slide; item Jaw 13.3) with NMI values around 0.25 and 0.26.

### 4.2 SHapley Additive exPlanations (SHAP) analysis

**FIGURE 2:**
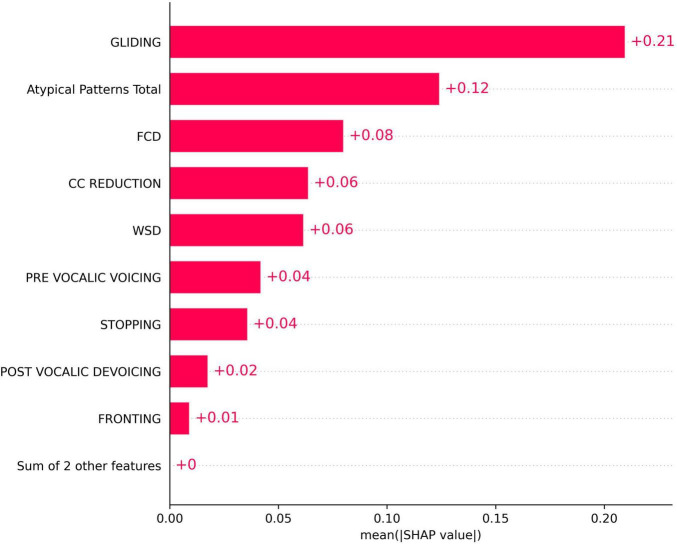
SHapley Additive exPlanations (SHAP) bar plot for the importance of phonological error patterns in predicting the total speech motor control error score.

Figure 2 presents the mean absolute SHAP values for each phonological error feature, quantifying their overall importance in the model. Among the features, GLIDING demonstrated the highest importance, with a mean SHAP value of approximately 0.21. Other features, including Atypical Patterns Total, FCD, and CC reduction, also contributed although with lower SHAP values. Features such as PRE-VOCALIC VOICING, POST-VOCALIC DEVOICING, and FRONTING showed relatively less affect.

**FIGURE 3:**
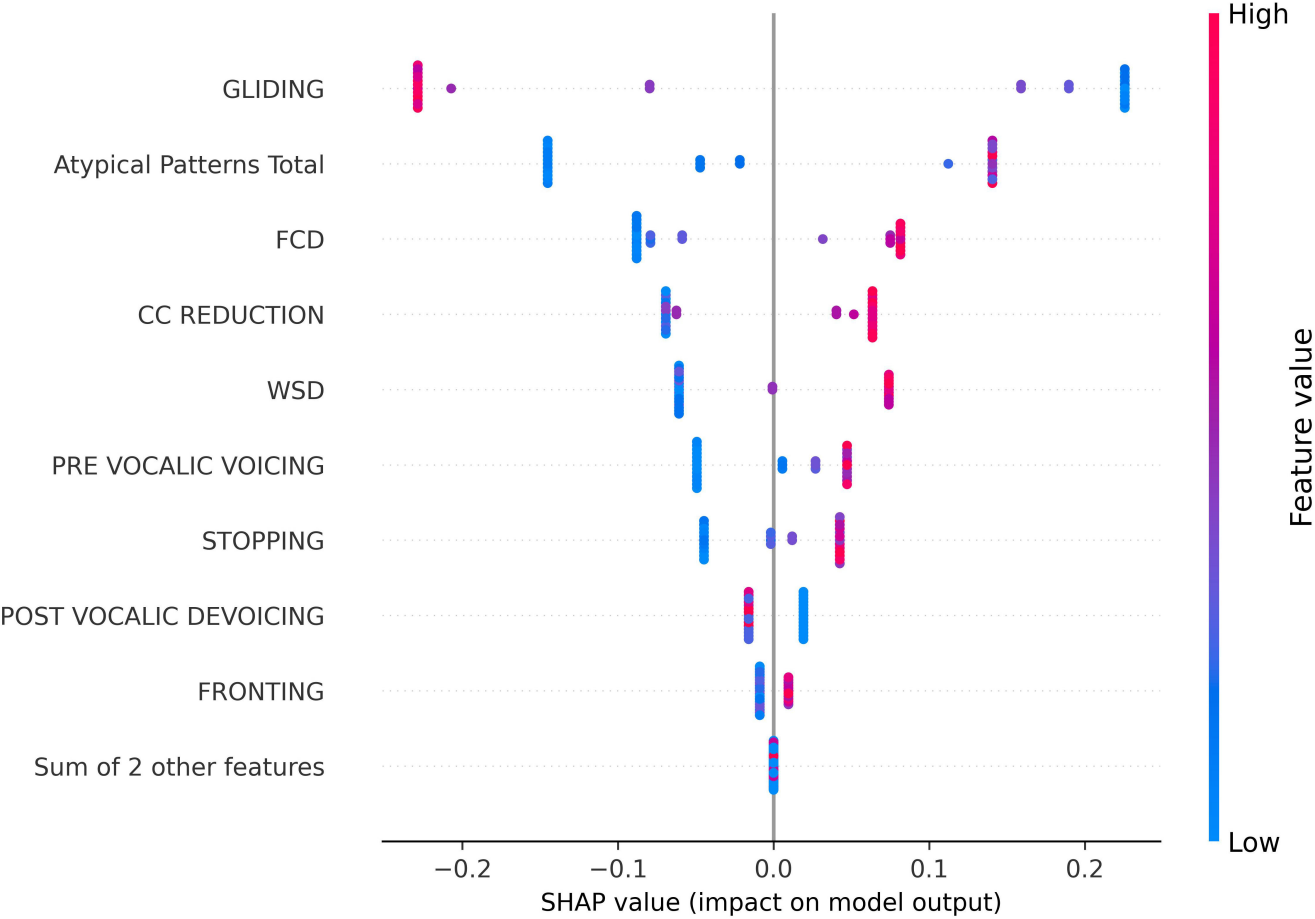
SHapley Additive exPlanations (SHAP) swarm plot for the impact of phonological error features on the predicted speech motor control score for all samples in the dataset.

The SHAP swarm plot (Figure 3) provides further insight into the directional influence of these phonological errors on model predictions. In this plot, the x-axis represents the SHAP values, indicating the magnitude and direction of a feature’s impact on the model’s output. Positive SHAP values suggest that the feature increases the predicted score, while negative values indicate a decreasing effect. The y-axis lists the phonological error features ranked by importance. Each dot corresponds to a single data sample, showing the SHAP value for that feature in that particular instance. The color gradient represents the feature value, with red indicating higher values and blue representing lower values.

Interestingly, as shown in Figure 3, the GLIDING error exhibited a negative impact on the prediction for most instances, which distinguished it from the other phonological features. This suggests that, higher gliding occurrences are typically associated with lower speech motor control error scores. In contrast, most other features, such as FCD, CC REDUCTION, and atypical patterns, generally displayed a positive association with the model output, meaning that increased occurrences of these errors tend to elevate the predicted score.

**FIGURE 4:**
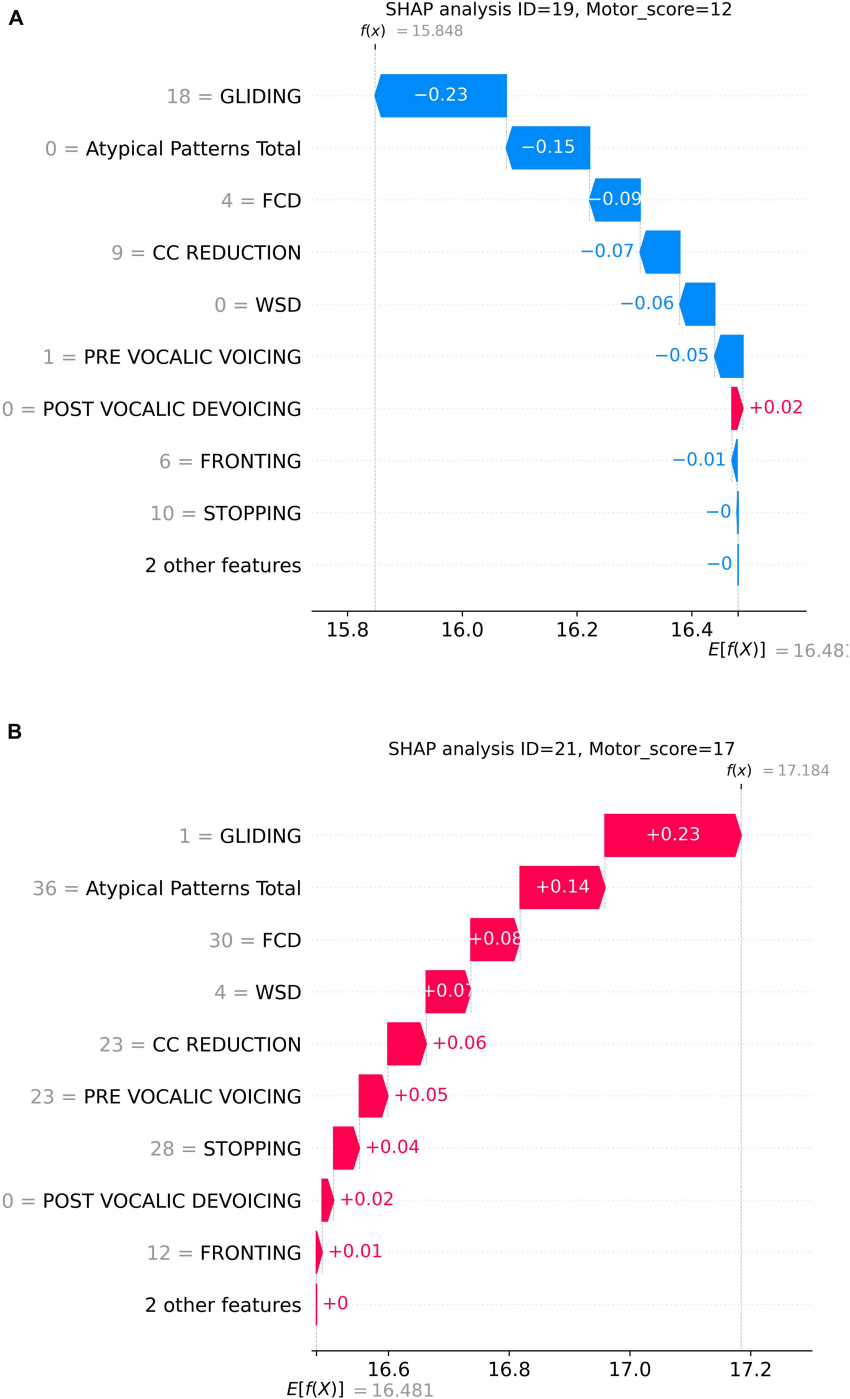
Prediction examples of two samples: **(A)** higher GLIDING error count and lower motor score; **(B)** lower GLIDING error count and higher motor score.

Figure 4 presents two specific prediction examples to demonstrate this counter-directional relation between GLIDING and total speech motor error score. Here, SHAP waterfall plots were employed to detail how individual phonological features contribute to the predicted speech motor control error score. In each graph, the x-axis represents the model’s predicted motor score *f* (*x*), with *E*[*f* (*x*)] presenting the average prediction across all samples. The y-axis lists the phonological features with their corresponding counts. Each bar represents the SHAP value for a feature, indicating how much it increases or decreases the prediction. In Figure 4A, the participant had a higher GLIDING error count of 18 and a lower total speech motor error score of 12. Thus, GLIDING played a role in reducing the predicted speech motor error score with a negative SHAP (−0.23). Meanwhile, a lower GLIDING count of 1, as shown in Figure 4B, presented a positive influence on the prediction with a SHAP value of +0.23. On the other hand, other phonological errors in both examples, such as FCD, CC REDUCTION, and Atypical Patterns, exerted the same direction influence on the predicted motor score, i.e., higher/lower phonological error counts increasing/decreasing the prediction.

Since gliding affects the later-acquired, complex /r/ and /l/ sounds, we expected higher motor speech checklist scores (i.e. more speech motor errors) to correlate with more gliding. However, contrary to our predictions, greater gliding was observed with lower speech motor control error scores. To further investigate, we examined whether gliding errors were more prevalent in older children and those with fewer speech motor issues compared to younger children or those with more severe speech motor control deficits. To assess this, we analyzed data from the demographics Table 1 (Namasivayam et al., 2021a; Namasivayam et al., 2021b), focusing on Percent Consonants Correct (PCC) as a measure of speech severity (>85% = mild, 65%–85% = mild-moderate, 50%–64% = moderate-severe, <50% = severe; DEAP test, Dodd et al., 2002) and speech motor scores from the standardized Verbal Motor Production Assessment for Children (VMPAC, Hayden and Square, 1999). The VMPAC systematically evaluates the neuromotor integrity of the speech motor system in children with speech sound disorders. For this study, we used the oromotor control and sequencing subsections of the VMPAC. Raw scores from these sections were divided by their total possible scores and converted into percentage scores (ranging from 0 to 100), where lower scores indicate poorer speech motor control, and higher scores reflect better speech motor performance.

**FIGURE 5:**
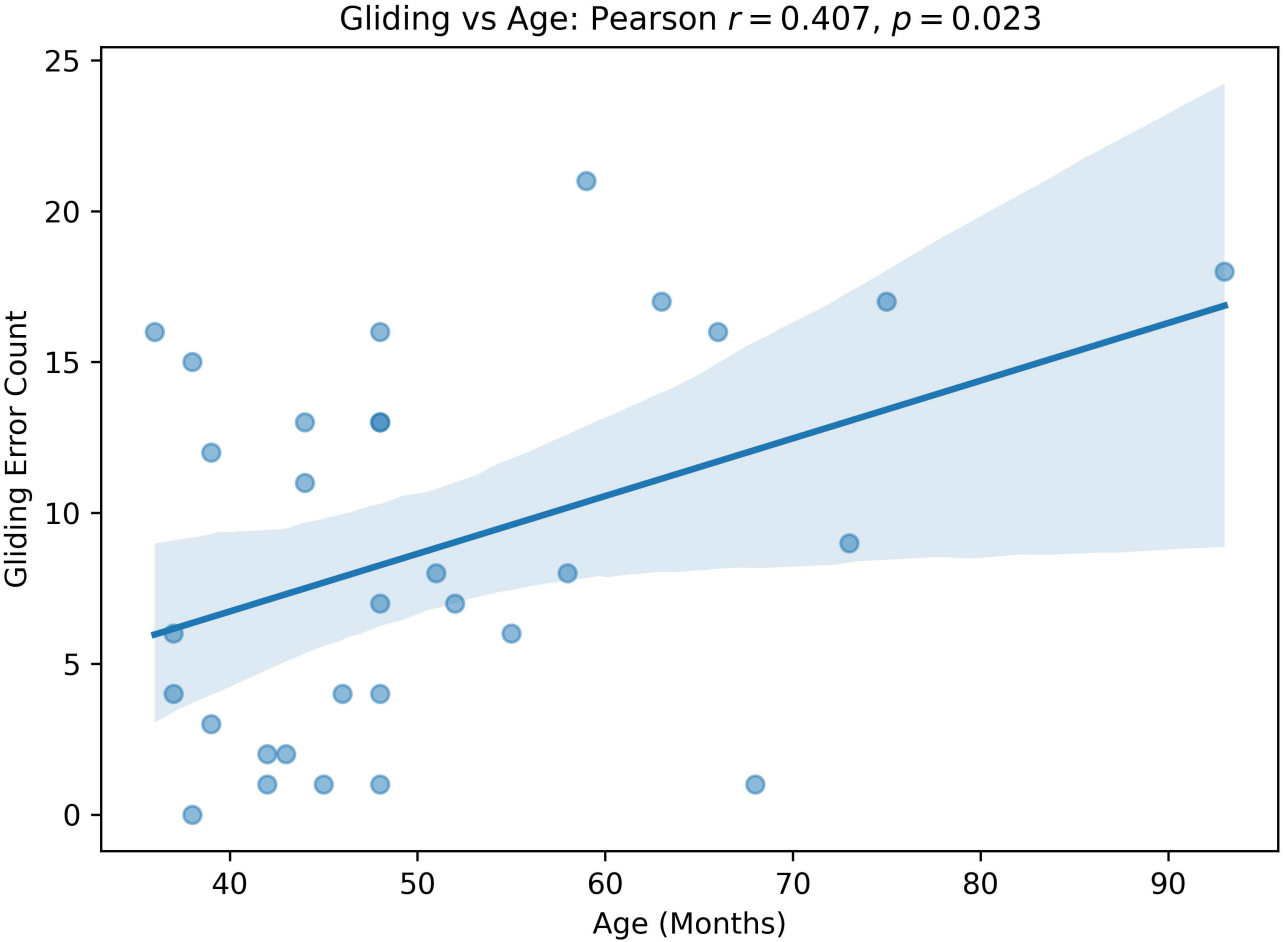
Pearson correlation and linear regression analysis between participant’s age (months, x-axis) and Gliding error count (y-axis).

**FIGURE 6:**
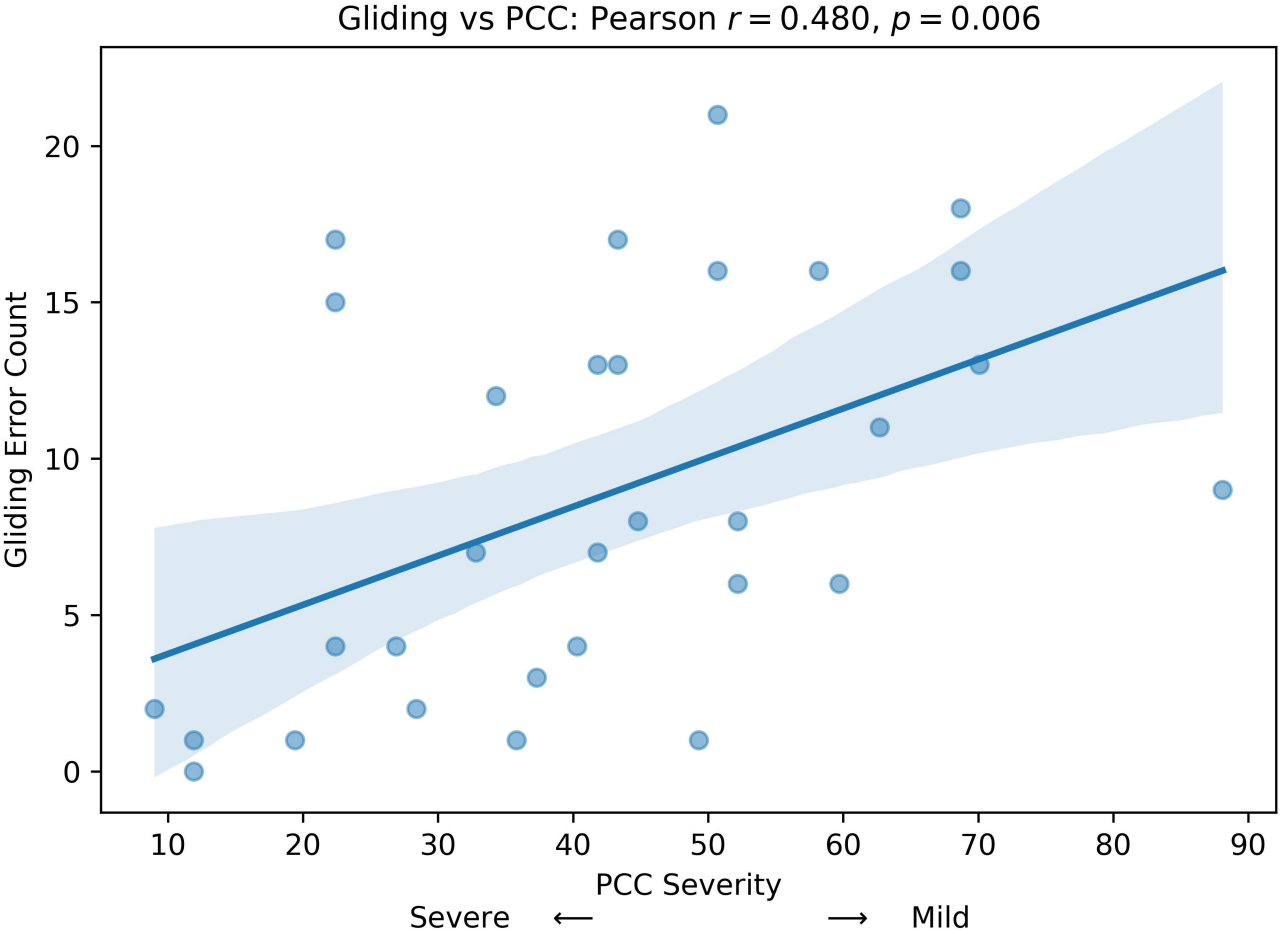
Pearson correlation and linear regression analysis between participant’s speech severity (percent consonants correct (PCC) score, x-axis) and Gliding error count (y-axis).

We computed Pearson correlation coefficients between GLIDING and both age in months and percent consonants correct (PCC), respectively, to assess whether gliding is more frequent in older or milder children. The results show a statistically significant positive relationship between Gliding vs. Age (*r* = 0.407, *p* = 0.023; Figure 5) and Gliding vs. speech severity (*r* = 0.480, *r* = 0.006; Figure 6). Also, the linear regression lines fitted with the data demonstrate significant positive slopes in both cases.

**FIGURE 7:**
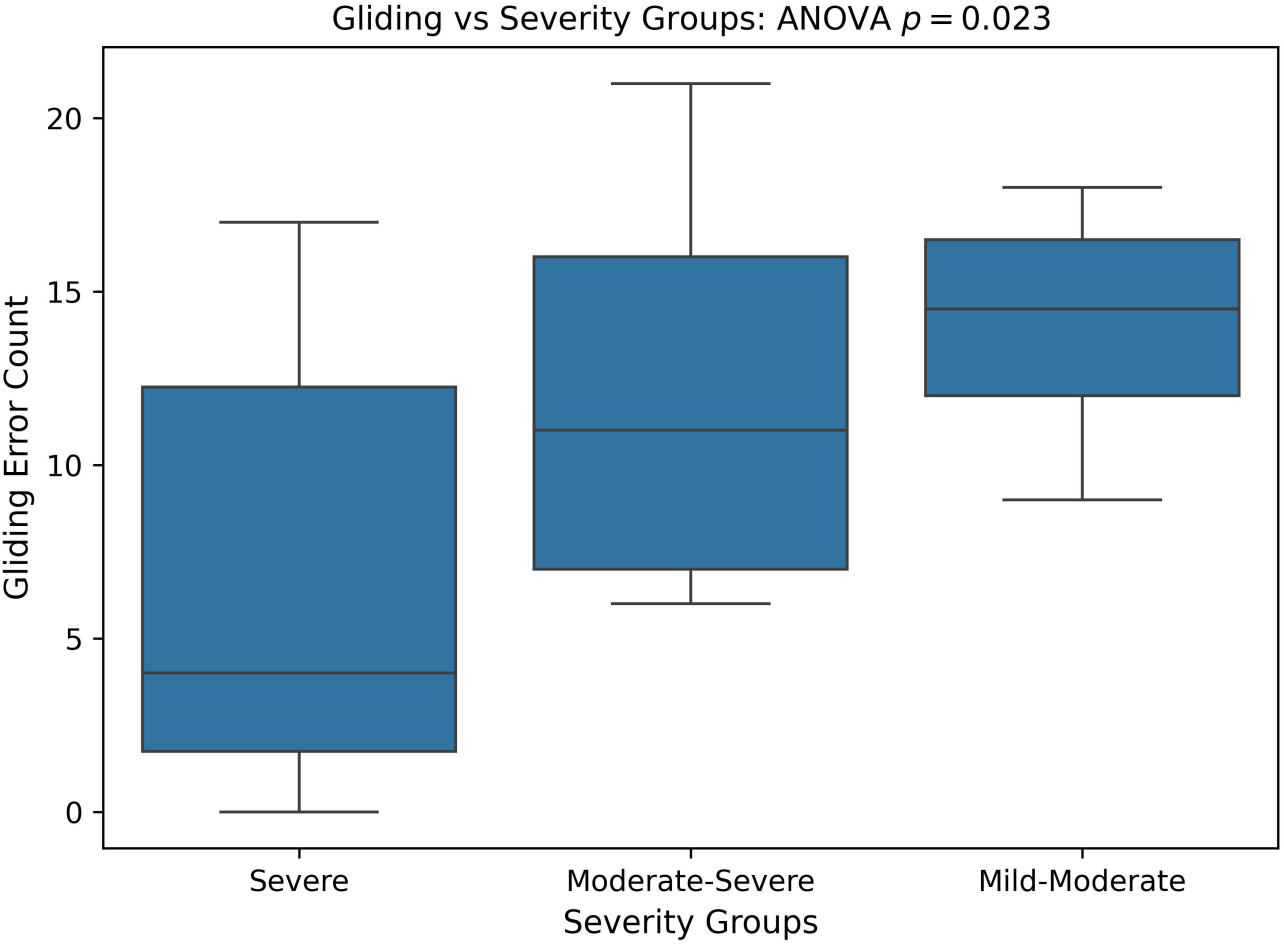
Boxplot and ANOVA analysis of Gliding error counts across different severity groups based on the percent consonants correct (PCC) score. PCC severity: severe (PCC < = 50), moderate-severe (50 < PCC < 65), and mild-moderate (PCC > = 65).

Next, we divided patients into groups based on PCC severity: severe (PCC < = 50), moderate-severe (50 < PCC < 65), and mild-moderate (PCC > = 65). As there was only one mild participant, the mild and mild-moderate groups were combined. A one-way ANOVA as well as a Kruskal-Wallis test was utilized to see if Gliding counts differ significantly across PCC severity groups. Note that Kruskal-Wallis test is a non-parametric version of the one-way ANOVA (no normality assumption). Figure 7 compares the distribution of gliding errors in these three PCC severity groups. There were significant differences in gliding errors of the three groups (ANOVA *p* = 0.023; Kruskal-Wallis *p* = 0.026).

**FIGURE 8:**
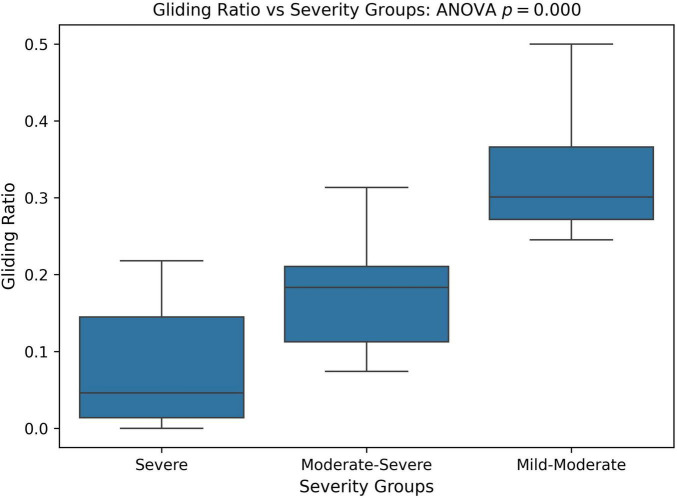
Boxplot and ANOVA analysis of Gliding error ratio across different severity groups based on the percent consonants correct (PCC) score. PCC severity: severe (PCC < = 50), moderate-severe (50 < PCC < 65), and mild-moderate (PCC > = 65).

We also compared the relative frequency of gliding errors to all phonological errors across different PCC severity groups. The goal was to determine whether the milder PCC group exhibited more gliding errors but fewer other phonological errors, while the severe PCC group had fewer gliding errors and more of other phonological error patterns. To assess this, we calculated the ratio of gliding errors to other phonological errors, referred to as the gliding ratio, across different PCC severity groups. Statistical significance was analyzed using one-way ANOVA and Kruskal-Wallis tests. As shown in Figure 8, the gliding ratio differed significantly between severity groups (ANOVA: *p* < 0.001; Kruskal-Wallis: *p* = 0.001).

Finally, we assessed the convergent validity of these findings by calculating Pearson correlation coefficients between gliding errors, total speech motor checklist scores, and the VMPAC standardized speech motor assessment (Hayden and Square, 1999). VMPAC scores were significantly negatively correlated with total speech motor checklist scores, *r*(30) = −0.45, *p* = 0.012, while they were significantly positively correlated with total gliding errors, *r*(30) = 0.59, *p* = 0.0006.

## 5 Discussion

The present study employed statistical approaches including Mutual Information Analysis and Random Forest Models (Breiman, 2001; Scott and Su-In, 2017; Strehl and Ghosh, 2002) to investigate the relationship between observed speech motor control characteristics (e.g., limited tongue tip elevation from the jaw) and the types of phonological errors identified in the DEAP phonological assessment (Dodd et al., 2002). The study involved a group of 48 preschool-aged children with moderate-to-severe SSDs (Namasivayam et al., 2021a; Namasivayam et al., 2021b). We hypothesized a non-zero (i.e., non-independent) relationship between these variables, suggesting that speech motor factors influence phonological error patterns.

Overall, the data analysis revealed that cluster reduction (CC REDUCTION), final consonant deletion (FCD), stopping, atypical patterns, and gliding were the most frequent phonological error patterns among participants, with mean frequencies ranging from 8.5 to 16.6 occurrences. In contrast, other phonological errors such as vocalization of liquids, deaffrication, and postvocalic devoicing occurred less frequently. The nature and distribution of phonological error patterns are in line with those reported in the literature for this population and age group (McLeod and Baker, 2017, Namasivayam et al., 2013). The distribution of speech motor control limitations was relatively uniform across participants, with an average total motor checklist score of 16.5.

The findings from both MI and SHAP analyzes provide converging evidence for the intricate interaction between speech motor control and phonological error patterns in children with moderate-to-severe SSDs. The MI analysis (Figure 1) revealed moderate associations between specific speech motor control limitations, particularly in labial-facial, lingual, and jaw control, and various phonological errors, such as final consonant deletion (FCD), gliding, and stopping. Notably, labial-facial control (items 14.1 and 14.2) and lingual control (items 16.1 and 16.2) exhibited the strongest associations with phonological errors (NMI > 0.30). The highest observed association was between FCD and labial-facial control (NMI = 0.47), reinforcing the idea that observed phonological error patterns may reflect underlying speech motor deficits. Similarly, atypical phonological errors (e.g., backing of anterior lingual sounds) showed a strong association with posterior movement-related lingual control (NMI = 0.46), highlighting the motoric basis of these less common error patterns. This association between atypical errors and poor lingual control has been reported in several instrument-based studies (e.g., Gibbon, 1999; Gibbon and Wood, 2002). Other motor domains, such as multi-plane movements and general speech production characteristics, demonstrated minimal relationships with phonological errors (NMI < 0.20). The MI analysis results indicate that phonological error patterns are not independent of speech motor control but are instead influenced by limitations in articulatory and speech motor control as proposed by Green et al. (2000, 2002) and others (e.g., Namasivayam et al., 2020), which challenges the traditional assumptions on the causal mechanisms underlying phonological error patterns (e.g., Dodd, 2014).

The SHAP analysis provided key insights into the contribution of specific phonological error patterns toward predicting speech motor control deficits, with GLIDING emerging as the most influential phonological feature (mean SHAP value ∼0.21). Interestingly, despite its strong contribution to model predictions, gliding demonstrated a negative directional influence, meaning that increased occurrences of gliding were associated with lower speech motor error scores. This pattern distinguished gliding from other phonological errors such as cluster reduction (CC reduction), final consonant deletion (FCD), and atypical patterns, which exhibited positive relationships with increased speech motor deficits. This unexpected relationship initially appeared counterintuitive, as the /r/ and /l/ sounds affected by gliding are complex, later-developing phonemes, theoretically associated with more mature speech motor control (McLeod and Baker, 2017). Further analysis, however, supported a motor-based explanation of gliding errors from a developmental and compensatory perspective. Specifically, correlation analyzes showed that gliding errors were significantly more frequent in older children and those with milder speech severity (higher PCC scores). Specifically, the milder PCC groups demonstrated significantly higher gliding counts and gliding-to-other-error ratios compared to severe groups. This pattern indicates that gliding, while often classified as a phonological error pattern, is intrinsically tied to speech motor maturation and articulatory complexity (Preston et al., 2020; Van Lieshout et al., 2008).

This interpretation is further supported by the significant negative correlation between standardized speech motor assessment (VMPAC; Hayden and Square, 1999) scores and speech motor checklist scores, alongside the positive correlation of VMPAC with gliding errors (*r* = 0.59, *p* < 0.001). These results reinforce that gliding correlates with relatively better speech motor capabilities rather than severe deficits. These findings suggest that gliding is prevalent among children who have developed sufficiently refined speech motor skills to attempt later-acquired, complex sounds but still experience difficulty coordinating multiple independent gestures (Preston et al., 2020; Van Lieshout et al., 2008). Electromagnetic articulography, electropalatography and ultrasound studies further support this motor-based interpretation, demonstrating that errors involving liquids often result from incomplete or poorly coordinated articulatory gestures (Adler-Bock et al., 2007; Gibbon, 1999; Gibbon and Wood, 2002; Gick and Campbell, 2003; Goozée et al., 2007; Preston et al., 2020; Van Lieshout et al., 2008). Thus, it is likely that children who are gradually acquiring these challenging articulations may temporarily resort to gliding as a compensatory simplification strategy due to the high demands of coordinating independent articulatory gestures (Namasivayam et al., 2020; Van Lieshout et al., 2008). These findings suggest that Gliding may represent an intermediate developmental stage in mastering challenging articulations.

While clinical psycholinguistic models, such as Dodd’s MDD (Dodd, 2014; Dodd et al., 1989), primarily attribute phonological error patterns to cognitive-linguistic or rule-based deficits rather than motoric influences, the present findings challenge this assumption by demonstrating systematic relationships between phonological error patterns and underlying speech motor control in the analysis of a sample of 30 preschool-aged children with moderate-to-severe SSDs. Crucially, the association we report is not unique to SMD subtype. Converging evidence indicates similar linkages between “phonological” errors and articulatory constraints in other SSD subtypes (e.g., persistent speech sound disorders, articulation and phonological disorders; Preston et al., 2020; Gibbon, 1999; Gibbon and Wood, 2002), in adults with neurological disorders (Hagedorn et al., 2017; Rong and Green, 2019), and even in typical adult speech under certain task demands or rate conditions (Browman and Goldstein, 1992; Goldstein et al., 2007). Thus, many patterns commonly labeled as phonological in children with SSD also emerge in typically developing children and in typical adults when articulatory dynamics are stressed, suggesting that these errors can arise from lawful interactions among gestural coordination, developing motor skill, and language-specific functional constraints (Browman and Goldstein, 1992; Goldstein et al., 2007; Namasivayam et al., 2020).

We acknowledge that this study did not directly assess cognitive resources (e.g., working memory) or speech-specific perceptual abilities (beyond a basic hearing screen), and thus we cannot fully exclude their contribution to the observed error patterns. That said, prior work by Shriberg and colleagues (Shriberg et al., 2010; Shriberg and Wren, 2019) has not consistently identified these factors as defining features of the SMD phenotype, which tempers though does not eliminate this concern.

Finally, observed associations between speech motor deficits and phonological error patterns provide support for theories such as Natural Phonology (Stampe, 1969), Grounded Phonology (Archangeli and Pulleyblank, 1994), and Articulatory Phonology (Browman and Goldstein, 1992; Namasivayam et al., 2020). These theories acknowledge that phonological processes are not purely cognitive-linguistic but are shaped by articulatory constraints. Taken together, these results underscore the necessity of integrating speech motor considerations into existing clinical models of speech sound disorders, advocating for a more comprehensive approach that bridges cognitive-linguistic and speech motor perspectives.

## 6 Conclusion

This study investigated the relationship between speech motor control deficits and phonological error patterns in preschool-aged children with moderate-to-severe SSDs using Mutual Information and SHAP analyses. Results demonstrated that phonological error patterns, especially cluster reduction, final consonant deletion, stopping, atypical patterns, and gliding, were systematically associated with speech motor limitations involving labial-facial, lingual, and jaw control. Notably, gliding errors showed an unexpected negative relationship with overall speech motor deficits, suggesting that gliding may represent a motor-based compensatory strategy rather than purely a phonological simplification. Specifically, gliding may represent an intermediate developmental stage in mastering challenging articulations. Overall, these findings challenge purely cognitive-linguistic/phonological explanations for patterns of speech sound errors in children and support integrative frameworks which emphasize the role of speech motor control and maturation in shaping observed speech sound error patterns.

## Data Availability

The datasets generated and/or analyzed during this study are not publicly available due to ethical restrictions. De-identified data are however available from the corresponding author upon reasonable request and with approval from the Research Ethics Board at the University of Toronto.
